# Identification of a new class of *WNT1* inhibitor: Cancer cells migration, G-quadruplex stabilization and target validation

**DOI:** 10.18632/oncotarget.6622

**Published:** 2015-12-15

**Authors:** Lien-Cheng Chang, Tsung-Chih Chen, Shiag-Jiun Chen, Chun-Liang Chen, Chia-Chung Lee, Shih-Hsiung Wu, Yun Yen, Hsu-Shan Huang, Jing-Jer Lin

**Affiliations:** ^1^ Institute of Biopharmaceutical Sciences, National Yang-Ming University, Taipei 112, Taiwan, ROC; ^2^ Food and Drug Administration, Ministry of Health and Welfare, Taipei 115, Taiwan, ROC; ^3^ Graduate Institute of Cancer Biology and Drug Discovery, College of Medical Science and Technology, Taipei Medical University, Taipei 110, Taiwan, ROC; ^4^ School of Pharmacy, National Defense Medical Center, Taipei 114, Taiwan, ROC; ^5^ Institute of Biological Chemistry, Academia Sinica, Taipei 115, Taiwan, ROC; ^6^ Institute of Biochemistry and Molecular Biology, National Taiwan University College of Medicine, Taipei 100, Taiwan, ROC

**Keywords:** Wnt1-mediated signaling pathway, G-quadruplex, 6-substituted 9-chloro-11H-indeno[1, 2-c]quinolin-11-ones, reporter assay

## Abstract

Developing the Wnt pathway inhibitors has been considered as a therapeutic approach for cancers and other Wnt-related diseases. Previously we found that the G-rich sequence of *WNT1* promoter is capable of forming G-quadruplex structure and stabilizing agents for Wnt1-mediated signaling pathway. Using a established cell-based drug screen system that enabled the evaluation of *WNT1* expression activity in a G-quadruplex structure dependent manner, we evaluated a series of 6-substituted 9-chloro-11H-indeno[1,2-*c*]quinolin-11-one derivatives that potentially inhibit the Wnt1-mediated signaling pathway. The most potent compound SJ26 showed repression of *WNT1* activity in a G-quadruplex structure-dependent manner. Moreover, compound SJ26 inhibited the *WNT1*-mediated downstream signaling pathway and suppressed migration activity of cancer cells. Thus, we have identified a tetracyclic azafluorenone, SJ26, that is capable of binding to G-quadruplex DNA structure, repressing *WNT1* expression, and inhibiting cell migration.

## INTRODUCTION

Tumor metastasis is the most common cause of cancer related-death. Targeting metastasis is considered as one of the highest priorities for researches in antitumor agents [1]. The Wnt-mediated signal pathway regulates various cellular functions, including proliferation, survival, migration, and development by trigger downstream signaling cascades [2]. Many of the Wnt-targeted genes are involved in tumorigenesis and metastasis. For example, *myc* and cyclin D1 are involved in proliferation and WISP1 is involved in angiogenesis [3, 4]. Wrch1 and *MMP7* are also known to be involved in migration and invasion of cancer cells [5, 6]. Clinically, aberrant activation of Wnt signaling is observed in a variety of tumors. Previous studies reported that high levels of *WNT1* expression in patients are associated with advanced metastasis [7–9]. and the overall survival is lower in patients with Wnt1-positive cancer. Thus, developing the Wnt pathway inhibitors has been considered as a therapeutic approach for the treatment of patients with cancers and other Wnt-related diseases [10, 11].

Small molecule inhibitors of the Wnt signaling pathways have been designed to target mediators of Wnt-signaling pathway [11]. These compounds mainly aim to decrease the levels of β-catenin [12–16]. Agents targeting Wnt directly have also been developed. For example, the anti-Wnt1 antibody was used to block the stimulation of Wnt1 downstream signaling pathway. Treatment of anti-Wnt1 antibody was shown to reduce the growth of hepatocellular carcinoma and colorectal cancer both *in vivo* and *in vitro* [17, 18].

Previous our study reported that the G-rich sequence of *WNT1* promoter is capable of forming both hairpin and G-quadruplex structures in the presence of potassium ion [19, 20]. Significantly, the Wnt1-mediated signaling pathway can be repressed upon the addition of G-quadruplex stabilizing agents in cancer cells. Consequently, the migration and invasion activities of cancer cells were also decreased [19]. Thus, it is likely that suppression of tumor metastasis can be achieved through stabilizing the G-quadruplex forming sequence located at the *WNT1* promoter.

**Figure 1:**
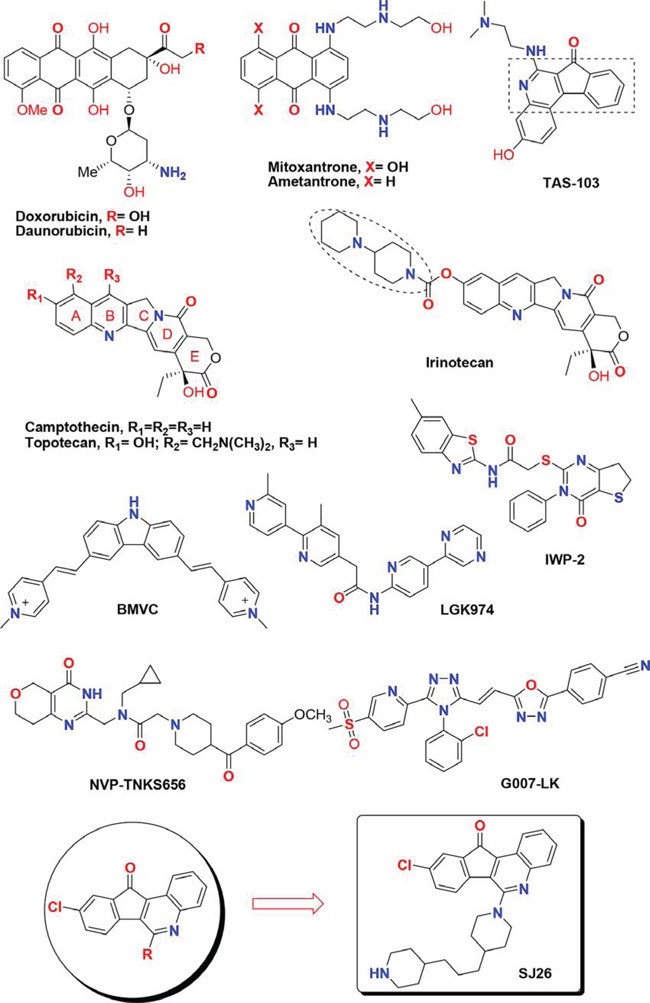
Chemical structures of several known tetracyclic quinoline derivatives, topoisomerase I inhibitors and Wnt modulators

Diverse anthracycline derivatives (e.g. doxorubicin, daunorubicin, mitoxantrone and ametantrone) have been shown to have anti-proliferative (or cytostatic) properties. We and others showed that the structurally related anthraquinone compounds can stabilize G-quadruplex structure formed by telomeric DNA sequences and inhibit telomerase or topoisomerase activity [21–33]. Camptothecin (CPT) and TAS-103 are also cytotoxic quinoline alkaloid derivatives that show potent topoisomerase (topo) I and/or topo II inhibition activities [34–37]. Two related CPT family members, irinotecan and topotecan, are currently used clinically as anticancer chemotherapy drugs [38, 39]. Based on the structures of anthracycline, here we design and synthesize a series of 6-substituted 9-chloro-11H-indeno[1,2-*c*]quinolin-11-one derivatives by varying the side chains with heterocyclic amine moieties and lipophilic alkyl chains (Figure 1). We have also integrated the pharmocophore of camptothecin (CPT) and TAS-103 into our drug design [40, 41]. The synthesis and results of these series of compounds against various cell lines and other SAR evaluation will be reported in a separate paper. In this study, the G-quadruplex structure-dependent *WNT1* repression activities of these newly synthesized compounds were analyzed by a cell-based assay system. We found that compound SJ26 showed potent *inhibitory effects* to the Wnt1-mediated downstream signaling pathway in a G-quadruplex structure dependent manner and inhibited the migration activity of cancer cells. Our results suggested the tetracyclic azafluorenones are potent *WNT1* repressors.

## RESULTS

### Cell-based system for expression repressors of *WNT1* gene

Functional analysis of human *WNT1* proximal promoter using reporter assays revealed that the 277-bp upstream sequence of *WNT1* is sufficient for the control of developmentally regulated expression [42, 43]. Sequence analysis of the 277-bp sequence identified two TATA boxes and a stretch of extremely G-rich sequence. Significantly, the G-rich sequence of the *WNT1* promoter contains four runs of at least three contiguous guanines that are capable of forming G-quadruplex structures under physiological conditions [19, 20].

**Figure 2:**
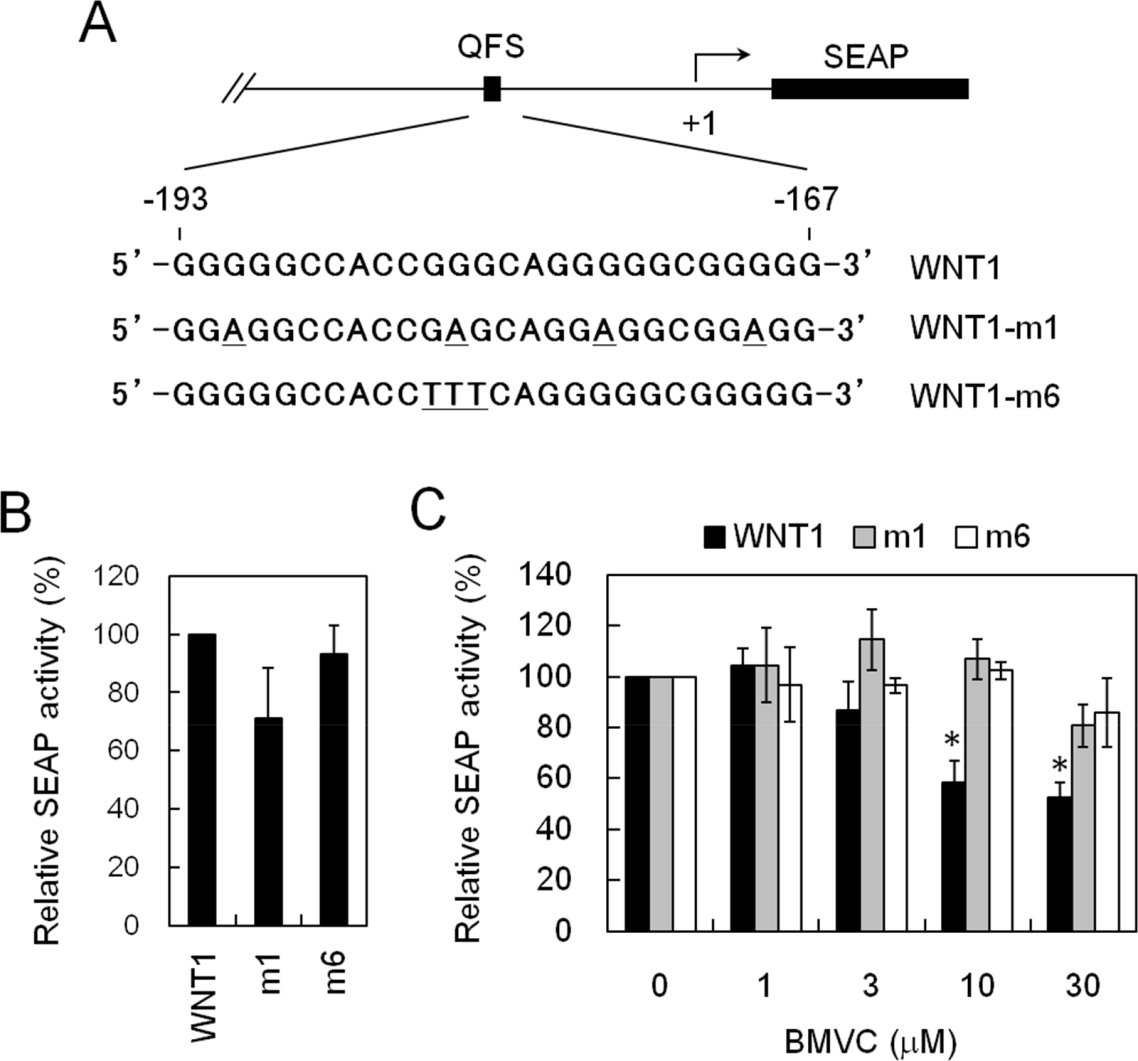
Establishing a cell-based assay system that inhibits *WNT1* expression through stabilizing the G-quadruplex structure formed at its promoter **A.** Schematic diagrams showed the mutation sites of *WNT1* in reporter assays. The G-quadruplex-forming sequences of Wnt1 (wild-type), Wnt1-m1, or Wnt1-m6 (Wnt1 mutations) were indicated. **B.** Wnt1-m1 and m6 mutations did not affect the basal expression level of *WNT1*. The H1299 cells harboring wild-type, WNT1-m1, and Wnt1-m6 reporters were analyzed for their basal expression activity. The relative phosphatase activities of wild-type, WNT1-m1, and WNT1-m6 mutant were presented using the wild-type level as 100%. **C.** BMVC repressed *WNT1* expression required G-quadruplex structure formation. The H1299 cells harboring wild-type, WNT1-m1, and WNT1-m6 reporters were incubated with the indicated concentrations of BMVC for 2 days. The phosphatase activities were then analyzed using the activity of DMSO-treated cells as 100%. Asterisks indicate *p* < 0.05.

To facilitate the analysis of *WNT1* expression, we ligated downstream to the *WNT1* promoter a reporter gene, SEAP, to generate a *WNT1* promoter-driven reporter construct, pWNT1-SEAP. We have also constructed two mutants that failed to form G-quadruplex structure, m1 and m6 (Figure 2A). The expression of SEAP can then be used as the criterion for the measurement of wild-type and mutant *WNT1* expression efficiency. Stable human lung carcinoma cell (H1299) lines carrying wild-type or mutant plasmids were selected. Although reporter analysis using transient transfection method to introduce reporter plasmids into cells produces better results in general, the approach is not suitable for drug screening because it needs additional steps for the analysis. These additional steps are prone to introduce variations in the screens. Moreover, transfection step requires additional reagents that are not economic for large-scale screens. Thus, stable clones were employed in drug screens.

The basal phosphatase activities under the control of wild-type or mutants promoters were first analyzed. We detected similar phosphatase activities for wild-type, the Wnt1-m1, and the Wnt1-m6 promoters, suggesting that the mutation did not affect the general transcriptional activity of the *WNT1* promoter (Figure 2B). To test if our reporter system is capable of differentiate G-quadruplex structure-mediated repression of *WNT1* expression, the SEAP expression was monitored in the presence of 3,6-bis(1-methyl-4-vinylpyridinium) carbazole diiodide (BMVC) because it was shown to bind and stabilize the G-quadruplex structure at *WNT1* promoter [19]. Upon treatment with BMVC, the expressions of SEAP from the wild-type promoter were reduced (Figure 2C). In contrast, the expression efficiency of mutant promoters did not respond to BMVC treatments. Thus, formation of G-quadruplex structure is required for suppressing *WNT1* expression by BMVC. This result indicated that our reporter construct is capable of monitoring the expression of *WNT1*. Moreover, addition of WNT1-m1 and -m6 mutation enables the evaluation of whether the repression requires G-quadruplex structure. Thus, the cells harboring pWNT1-SEAP or its mutants could be used as a tool to monitor the expression of *WNT1* in a G-quadruplex structure dependent manner.

### Inhibitory effect of compound SJ26 on the *WNT1* expression of G-quadruplex dependent manner

The expression of SEAP in H1299 cells harboring these reporters were used as the criterion to evaluate if the 6-substituted 9-chloro-11H-indeno[1,2-*c*]quinolin-11-one derivatives repressed the expression of *WNT1* in cancer cells. To facilitate effective identification of compounds that selectively repress *WNT1* activity, we established a screening strategy that was adopted and modified from the NCI-60 Human Tumor Cell Lines Screen (https://dtp.cancer.gov/discovery_development/nci-60/methodology.htm). The relative *WNT1* expressions by these compounds at one-dose (10 μM) were first determined in these cells. We reasoned that an effective inhibitor should give good inhibitory effect at this concentration. The numbers reported (values between 0 and 100) for the one-dose assays represented the *WNT1* promoter activities relative to the no-drug control that were determined by SEAP assays (Table 1). To rule out compounds that showed apparent SEAP repression activities by inhibiting cell growth, MTT assays were also introduced into our screen. It is to eliminate compounds that showed *WNT1* repressing activities non-selectively. Compounds that exhibited significant *WNT1* promoter inhibition and did not show grow inhibitory activity in the one-dose screen are selected and further evaluated.

**Table 1:** Effect of tetracyclic azafluorenone derivatives on repressing *WNT1* expression

Compd SJ	R	H1299^a^					
		SEAP (% ± SD)^b^			MTT (% ± SD)		
		Wnt1	m1^c^	m6	Wnt1	m1	m6
**2**	−Cl	94.7 ± 34.9	96.22 ± 21.4	157.8 ± 20.6	114.5 ± 3.0	101.0 ± 17.2	132.9 ± 7.5
**3**	−NHCH_3_	17.1 ± 10.0	49.0 ± 10.8	32.6 ± 17.4	92.9 ± 4.9	139.2 ± 20.5	90.9 ± 5.5
**4**	−N(CH_3_)_2_	17.4 ± 9.1	79.4 ± 14.9	35.3 ± 21.5	101.2 ± 2.3	137.5 ± 16.2	83.8 ± 3.2
**5**	−NHCH_2_CH_2_N(CH_2_CH_3_)_2_	23.0 ± 2.4	25.7 ± 12.4	45.5 ± 26.3	97.1 ± 4. 6	113.9 ± 18.9	76.8 ± 5.5
**6**	−N(CH_2_CH_2_)_2_	33.3 ± 11.5	76.5 ± 18.6	34.2 ± 21.2	104.9 ± 1.0	102.9 ± 6.6	81.9 ± 8.3
**7**	−N(CH_2_CH_2_)_2_CH_2_	34.0 ± 14.7	75.1 ± 19.8	53.6 ± 15.7	113.8 ± 11.1	151.1 ± 1.0	92.0 ± 14.7
**8**	−N(CH_2_CH_2_)_2_CHCH_3_	57.6 ± 7.1	98.7 ± 31.1	58.1 ± 17.4	105.3 ± 10.9	163.8 ± 7.0	104.3 ± 14.1
**9**	−N(CH_2_CH_2_CH_2_)_2_	55.8 ± 13.9	85.8 ± 37.0	57.4 ± 15.8	118.2 ± 7.0	148.7 ± 1.0	96.6 ± 6.8
**10**	−N(CH_2_CH_2_)_2_O	30.1 ± 9.5	79.1 ± 18.1	46.7 ± 15.5	98.0 ± 9.0	119.6 ± 16.4	95.9 ± 3.6
**11**	−N(CH_2_CH_2_)_2_S	55.7 ± 14.7	115.5 ± 21.9	53.8 ± 13.4	109.0 ± 5.6	154.2 ± 15.2	110.8 ± 3.5
**12**	−N(CH_2_CH_2_)_2_NH	76.2 ± 13.4	103.5 ± 4.8	63.5 ± 13.1	88.8 ± 13.1	131.6 ± 23.1	105.4 ± 9.0
**13**	−N[CH(CH_3_)CH_2_](CH_2_CH_2_)NH	60.7 ± 0.9	115.3 ± 2.8	64.6 ± 3.0	120.1 ± 8.3	161.0 ± 0.3	100.3 ± 9.2
**14**	−N(CH_2_CH_2_)_2_NCH_3_	73.1 ± 7. 9	64.7 ± 22.1	50.1 ± 19.1	99.8 ± 1.2	145.3 ± 3.7	92.4 ± 1.1
**15**	−N(CH_2_CH_2_)_2_NCH_2_CH_3_	78.7 ± 11.4	110.3 ± 9.8	48.6 ± 17.0	112.5 ± 0.0	153.6 ± 21.0	113.8 ± 5.2
**16**	−N(CH_2_CH_2_)_2_NCH(CH_2_CH_2_)_2_	57.3 ± 14.0	131.8 ± 30.4	76.3 ± 7.8	121.0 ± 6.6	169.7 ± 14.0	120.0 ± 4.6
**17**	−N(CH_2_CH_2_)_2_CHN (CH_2_CH_2_)_2_CH_2_	38.4 ± 19.5	20.8 ± 14.0	51.3 ± 19.6	112.9 ± 3.9	157.8 ± 8.3	115.8 ± 9.8
**18**	−N(CH_2_CH_2_)_2_NC_6_H_5_	34.5 ± 13.8	49.4 ± 7.8	74.2 ± 12.9	98.5 ± 9.3	152.5 ± 6.9	94.6 ± 0.6
**19**	−N(CH_2_CH_2_)_2_NCH_2_C_6_H_5_	52.8 ± 11.9	101.6 ± 7.2	59.2 ± 4.2	140.37 ± 0.0	155.1 ± 6.2	111.2 ± 2.2
**20**	−N(CH_2_CH_2_)_2_NC_6_H_4_F(*o*)	82.5 ± 11.9	94.7 ± 16.9	66.4 ± 2.6	119.9 ± 9.0	145.8 ± 8.2	122.7 ± 5.1
**21**	−N(CH_2_CH_2_)_2_ NC_6_H_4_OCH_3_ (*o*)	24.7 ± 8.5	39.5 ± 11.2	63.3 ± 7.0	128.9 ± 16.7	165.3 ± 15.9	118.8 ± 17.4
**22**	−N(CH_2_CH_2_)_2_ NC_6_H_4_OCH_3_ (*m*)	47.1 ± 0.6	79.1 ± 8.6	63.9 ± 6.8	98.6 ± 4.4	110.7 ± 10.7	102.1 ± 11.4
**23**	−N(CH_2_CH_2_)_2_NCH (CH_2_CH_2_)_2_NCH_3_	49.2 ± 22.4	112.2 ± 26.7	55.6 ± 14.2	110.5 ± 3.2	135.6 ± 3.1	103.9 ± 12.1
**24**	−N(CH_2_CH_2_)_2_C(OCH_2_)_2_	54.8 ± 19.0	122.0 ± 12.8	65.4 ± 4.7	103.1 ± 1.9	131.0 ± 34.2	114.6 ± 4.3
**25**	−N(CH_2_CH_2_)_2_N(CO)N (CH_2_CH_2_)_2_CH_2_	65.7 ± 27.6	113.2 ± 20.7	63.5 ± 4.4	98.2 ± 14.9	146.5 ± 7.6	114.0 ± 6.8
**26**	−N(CH_2_CH_2_)_2_CH(CH_2_)_3_ CH(CH_2_CH_2_)_2_NH	42.7 ± 1.9	99.6 ± 22.7	83.7 ± 15.7	88.3 ± 8.5	127.6 ± 0.4	98.4 ± 5.2
**27**	−SCH_2_CH_2_OH	42.0 ± 7.6	96.8 ± 14.3	63.4 ± 1.7	97.0 ± 10.8	127.4 ± 12.9	79.1 ± 9.3

a Non-small lung cancer cells H1299 were grown in RPMI 1640 media supplemented with 10% fetal bovine serum, 100 units/mL penicillin and 100 mg/mL streptomycin in a humidified atmosphere with 5% CO_2_ at 37°C.

b SD, standard derivation; all experiments were independently performed at least three times.

c The G-quadruplex-forming sequences of WNT1 (wild-type), WNT1-m1, or WNT1-m6 (WNT1 mutations) were indicated.

**Figure 3:**
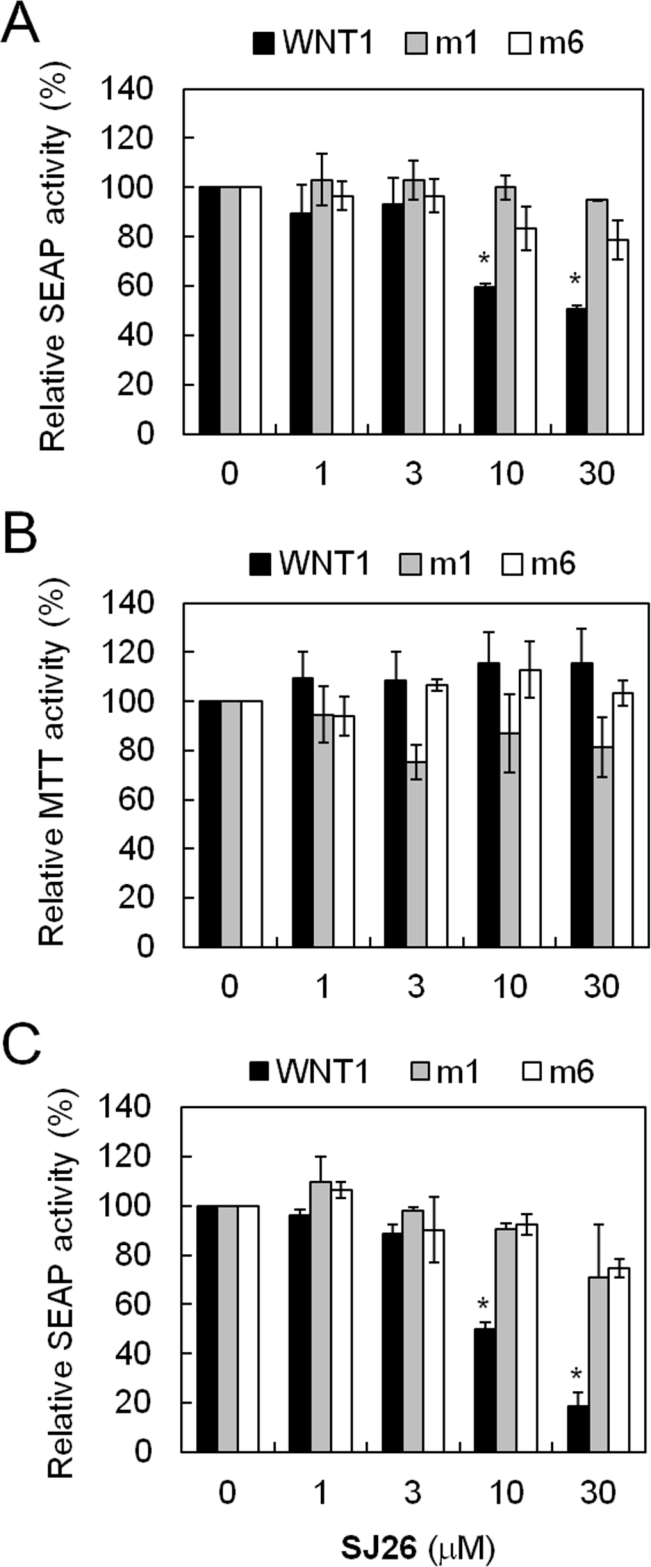
SJ26 repressed *WNT1* expression in a G-quadruplex dependent manner **A.** Suppression of *WNT1* expression by SJ26 using reporter-harboring stable cell lines. The H1299 cells harboring wild-type, WNT1-m1, and WNT1-m6 reporters were incubated with the indicated concentrations of SJ26 for 24 h. The phosphatase activities were then analyzed using the activity of DMSO-treated cells as 100%. **B.** Cytotoxic effects of SJ26. About 2 × 10^4^ H1299 cells were seeded in 96-well plates and incubated with SJ26 at various concentrations for 24 h. Cell growth was then determined using the MTT assay. The values are obtained from three experiments using the values of untreated cells as 100%. **C.** Suppression of *WNT1* expression by SJ26 using transient-transfected reporter assays. Reporter plasmid constructs carrying wild-type, WNT1-m1, and WNT1-m6 promoters were transfected into H1299 cells using lipofecamin 2000. The transfected cells were incubated with the indicated concentrations of SJ26 for 24 h. The phosphatase activities were then analyzed using the activity of DMSO-treated cells as 100%. Asterisks indicate *p* < 0.05.

As shown in Table 1, more than half [13 out of 25 including: **SJ3** (17.1%), **SJ4** (17.4%), **SJ5** (23.0%), **SJ6** (33.3%), **SJ7** (34.0%), **SJ10** (30.1%), **SJ17** (38.4%), **SJ18** (34.5%), **SJ21** (24.7%), **SJ22** (47.1%), **SJ23** (49.2%), SJ26 (42.7%), and **SJ27** (42.0%)] of the tested compounds repressed more than 50% of the SEAP activity in wild-type *WNT1* promoter, suggesting that this series of analogs are potent repressors of *WNT1* expression. At 10 μM concentration, none of the synthesized derivatives showed potent cytotoxicity, indicating that the observed *WNT1* repressing activities were not due to cellular toxicity (Table 1). To identify compounds that repressed *WNT1* expression through the formation of G-quadruplex structure at its promoter, the SEAP activity of wild-type, m1 and m6 mutants were next compared (Table 1). Among the tested compounds, only SJ26 did not repress m1 and m6 mutants, suggesting that the observed repression in wild-type promoter is G-quadruplex structure-dependent. We tested the SEAP repressing activity and cytotoxicity using multiple doses of SJ26. Consistent with the screening results, SJ26 selectively repressed wild-type *WNT1* promoter activity and the repression is G-quadruplex dependent as it did not significantly affect the other two mutant promoters (Figure 3). It is interesting to note that SJ26 did not show significant cytotoxic activity at concentration up to 30 μM. In addition to the stable lines that we used in the screen (Figure 3A and 3B), we have also conducted the reporter assays using transient transfection method to introduce reporter constructs into H1299 cells. As shown in Figure 3C, SJ26 selectively repressed the wild-type promoter, further confirming that the observed *WNT1*-repressing activity by SJ26 was mediated through the formation of G-quadruplex structure formed at the *WNT1* promoter. It is also apparent that transient transfect method yielded better inhibitory results.

### Effect of SJ26 on the melting temperature of *WNT1* G-quadruplex

**Figure 4:**
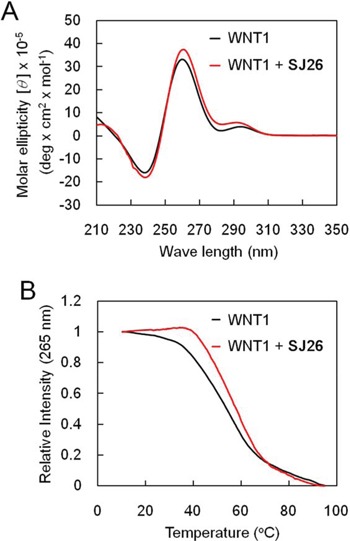
SJ26 enhances the melting temperature of *WNT1* G-quadruplex **A.** CD spectra of the *WNT1* G-rich sequence. The CD spectra of 100 μM WNT1 (5′-GGGGGCCACCGGGCAGGGGGCGGGGG-3′) oligonucleotide in the presence of 5 mM KCl were recorded over the spectral range of 210–350 nm. **B.** Temperature-dependent CD signals at 265 nm of 5 μM *WNT1* quadruplex and on interaction with 3 μM SJ26 were measured, respectively.

We have previously showed that an oligonucleotide carrying the G-rich sequence of *WNT1* promoter was capable of forming a G-quadruplex structure [19, 20]. To evaluate the effect of SJ26 to the G-quadruplex structure of *WNT1* promoter, we applied circular dichroism (CD) to monitor the melting temperature (Tm) of *WNT1* G-quadruplex. Oligonucleotide containing the G-quadruplex-forming sequences of *WNT1* promoter (−193 to −167) were synthesized and analyzed by CD spectormetry. The CD spectrum of *WNT1* oligonucleotide displayed a positive band at 264 nm and a negative band at 240 nm together with a relative weak positive band at 292 nm in the presence of 5 mM K^+^ (Figure 4A). The result is consistent with our previous observation that *WNT1* oligonucleotide forms a mixed parallel/antiparallel folding pattern with at least two different G-quadruplex conformations [19, 20]. Addition of SJ26 did not greatly affect the overall CD spectrum of the *WNT1* G-quadruplexes. The 265-nm CD band was then measured as a function of temperature to determine the Tm of *WNT1* G-quadruplexes in the presence of SJ26. Here we used relatively low molar ratio of SJ26 (3 μM) to *WNT1* DNA (5 μM) in our analysis. High molar ratio of SJ26 was not used because we consider that high molar ratio of SJ26 relative to its target DNA is not the pharmacologically equivalent concentration. We found that the Tm of *WNT1* G-quadruplexes was elevated from 54.2 to 57.7°C in the presence of 3 μM SJ26, suggesting that it could thermally stabilize the *WNT1* G-quadruplexes (Figure 4B).

### Effect of SJ26 represses *WNT1* expression and reduces the migration activity of H1299 cells

**Figure 5:**
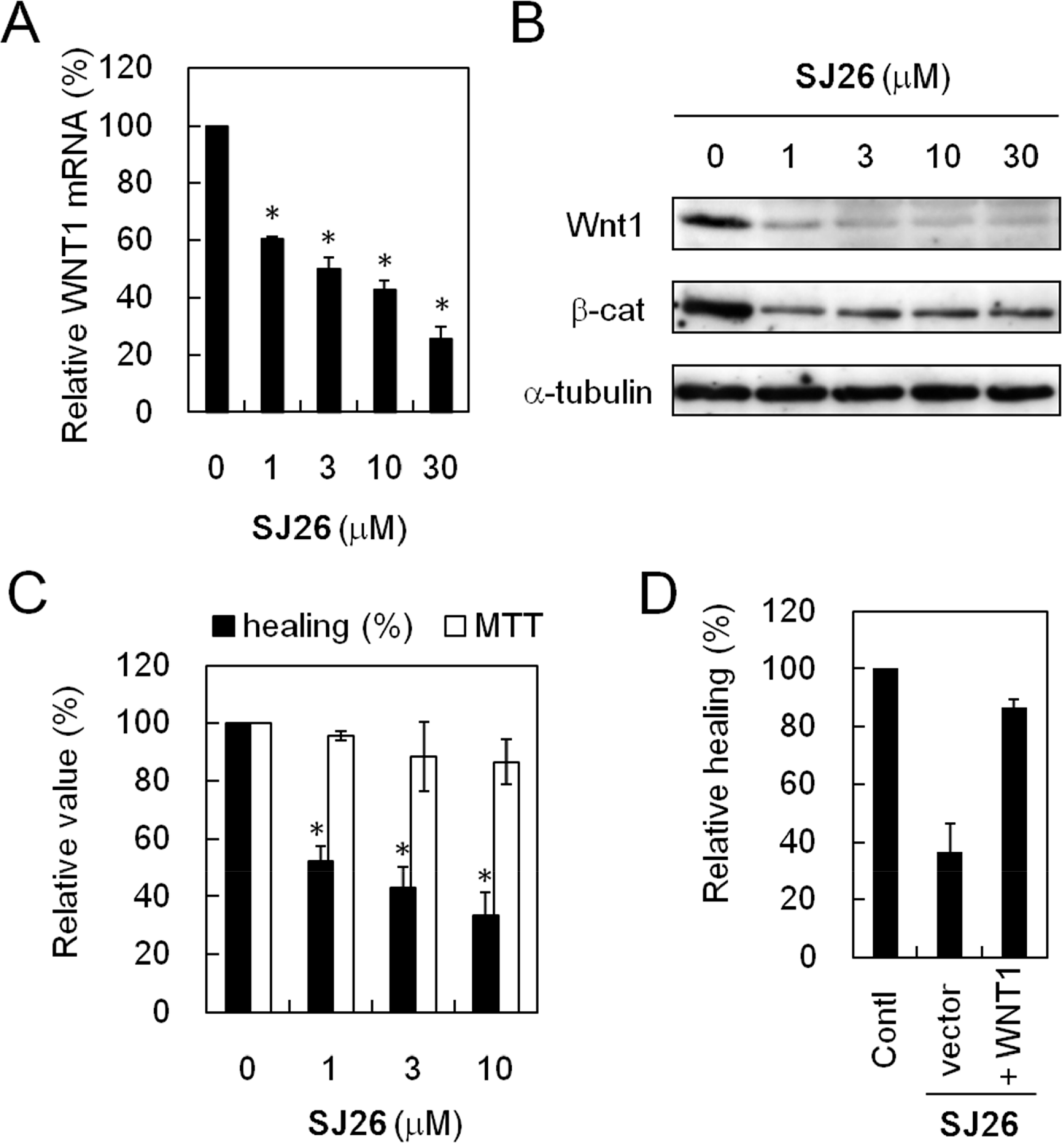
SJ26 repressed *WNT1* expression and reduced the migration activity of H1299 cells **A.** SJ26 reduced the *WNT1* mRNA level. The H1299 cells was incubated with 1, 3, 10, or 30 μM SJ26 for 24 h and then subjected to quantitative RT-PCR analysis. The GAPDH expression level was used as an internal control. The mRNA level of *WNT1* expression in untreated H1299 cells was defined as 100%. Results were obtained from the average of three independent experiments. Asterisks indicate *p* < 0.05. **B.** SJ26 reduced the Wnt1 protein and its down strean mediator β-catenin levels. H1299 cells were incubated with 1, 3, 10, or 30 μM SJ26 for 24 h. Total cell extracts were prepared, and immunoblotting analysis was conducted using antibodies against Wnt1, β-catenin, or α-tubulin. **C.** SJ26 reduced the healing activity of H1299 cells. H1299 cells were incubated with 10 μM DMSO or 1, 3, or 10 μM SJ26 for 48 h, and then the treated cells were subjected for scratch assays. Relative migration rates of H1299 cells were determined by migration distance over the time. Cell growth was also determined using the MTT assay. The value of migration rate or cell growth in DMSO-treated cells was defined as 100%. Asterisks indicate *p* < 0.05. **D.** *WNT1*-overexpression reversed the healing-inhibitory activity of SJ26. The relative healing rates were determined in 10 μM SJ26-treated cells that overexpressing *WNT1*. The relative healing rate in DMSO-treated H1299 cells was defined as 100%.

The effect of SJ26 on *WNT1* expression was further analyzed. As shown in Figure 5, real time RT-PCR analysis showed that the *WNT1* mRNA level was significantly decreased in cells that were treated with SJ26 (Figure 5A). Moreover, immunoblotting analysis of the SJ26-treated lung cancer H1299 cells showed that the Wnt1 protein levels were greatly decreased by SJ26 treatment at a dose as low as 1 μM (Figure 5B). These results indicate that SJ26 could stabilize G-quadruplex structure and reduce the expression of *WNT1*. It is also interesting to note that although it requires ∼10 μM to observed the repressing effect in our cell-based SEAP reporter system, SJ26 at concentration as low as 1 μM was able to suppress the endogenous *WNT1* expression. Although the reason is not clear to us, the apparent differences might be due to the difference of sensitivity in these assay systems. The downstream effect of Wnt1-mediated signaling pathway by SJ26 was also analyzed. Immunoblotting analysis showed that the β-catenin levels were decreased by SJ26 (Figure 5B). Since the Wnt1-mediated signaling pathway was shown to have an important role in cancer migration and invasion, the cellular effects of suppressing *WNT1* expression by SJ26 were analyzed. The migration activity of SJ26-treated cells was first evaluated using scratch assays. As shown in Figure 5C, the healing activity of H1299 cells was decreased by SJ26 in a dose dependent manner. The anti-proliferation efficacy of SJ26 was also determined. We found SJ26 did not affect the proliferation of H1299 cells, suggesting the observed healing-inhibitory effect was not due to inhibition of proliferation. To show that the anti-healing effect of SJ26 was through suppression of Wnt1-mediated pathway, we overexpressed *WNT1* in SJ26-treated cells. We expected that expressing of excess amount of Wnt1 should reverse the anti-healing effect by SJ26. Plasmid construct harboring *WNT1* under the control of a CMV promoter was transfected into H1299 cells. Indeed, the scratch analysis also indicated that the inhibitory effect of SJ26 was reversed in *WNT1*-overexpressing cells (Figure 5D). The results indicate that the migration inhibitory effect of SJ26 was mediated through suppressing the Wnt1-mediated signaling pathway.

### Suppression of Wnt1-mediated cell migration by SJ26 was not limited to H1299 cells

**Figure 6:**
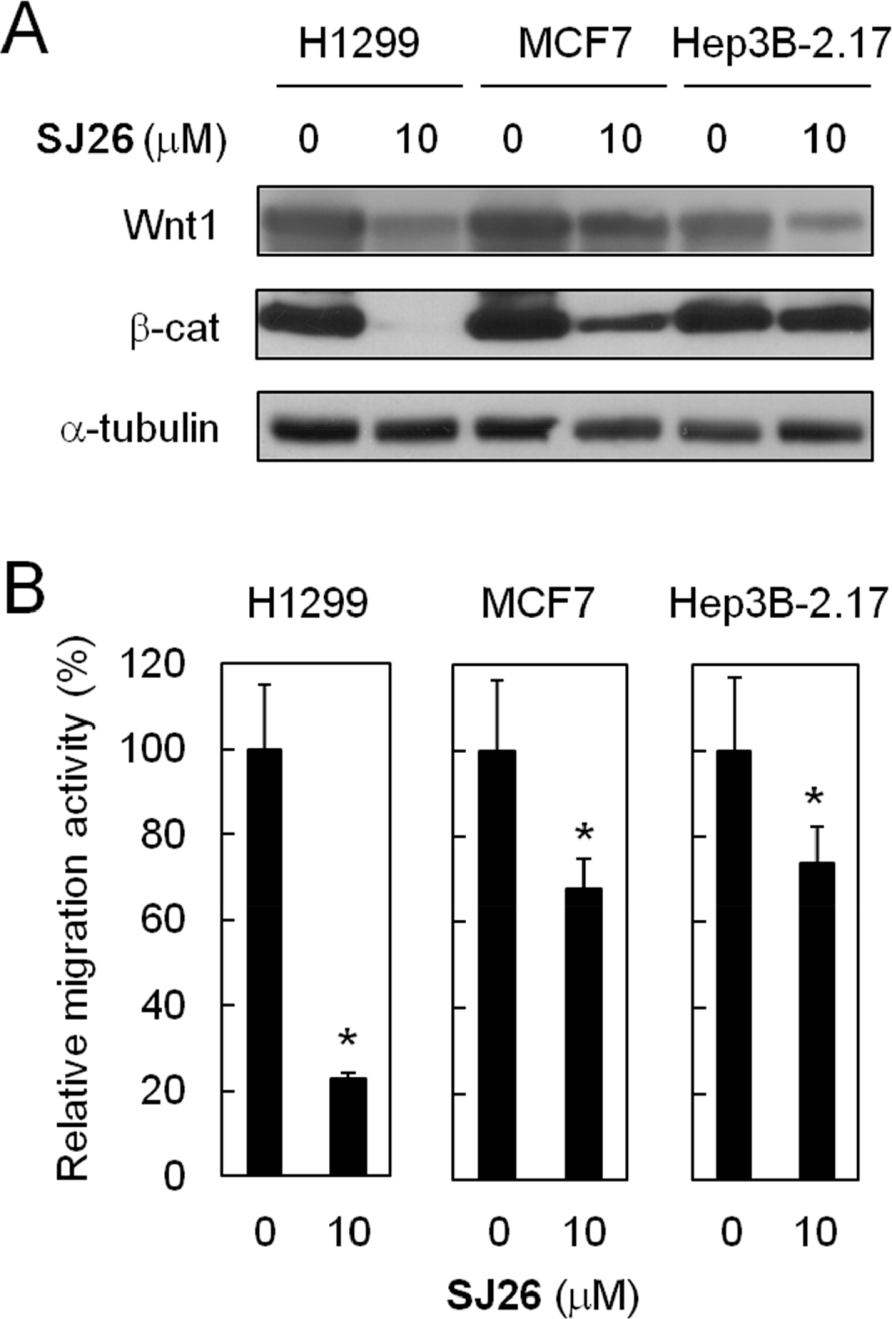
SJ26 suppressed cell migration in H1299, MCF7 and Hep3B 2.17 cells **A.** SJ26 suppressed Wnt1/β-catenin-signaling pathway in H1299, MCF7 and Hep3B 2.17 cells. Cells were treated with 10 μM SJ26 and the total cell extracts were prepared. Immunoblotting analysis was conducted using antibodies against Wnt1, β-catenin, or α-tubulin. **B.** H1299, MCF7 or Hep3B 2.17 cells were incubated with 10 μM of SJ26 and were subjected to trans-well assay analysis. The extent of cell migration across the wells was recorded after 6 hours. The value of migration rate in DMSO-treated cells was defined as 1. Asterisks indicate *p* < 0.05.

To test the effects of *WNT1* expression and migration upon SJ26 treatments on other cancer cell lines, the MCF7 (breast cancer) and Hep2B 2.17 (liver cancer) cells were also tested. We found SJ26 effectively repressed the expression of *WNT1* and β-catenin in these cells (Figure 6A). The migration activity was next evaluated using trans-well assays. As shown in Figure 6B, SJ26 effectively inhibited the migration activity of H1299 cells. Both the migration activities of MCF7 and Hep3B cells were also inhibited by SJ26, although not as effective as in H1299 cells. It was noted that the level of Wnt1/β-catenin repression by SJ26 correlated with their reduction in migration activity. Thus, the cellular context in different cell types might also have a role in modulating the repressing effects of SJ26. Nevertheless, the correlation between Wnt1/β-catenin repressing and migration inhibition by SJ26 provides strong indication that SJ26 inhibit the migration activity of cancer cells through repressing the expression of *WNT1*.

## DISCUSSION

Aberrant activation of the Wnt pathway is implicated in driving the formation of various human cancers. Many cancers show evidence of inappropriate activation of the Wnt pathway, including raised levels of β-catenin [44, 45]. Moreover, defective Wnt signaling might also play a role in the generation and maintenance of cancer stem cells [46]. Thus, Wnt signaling plays a pivotal role in cancer, and compounds that dampen Wnt pathway activity could provide useful molecular targeted therapeutics for the treatment of cancers [11, 47–49]. An assortment of small molecule inhibitors has been identified that target the mediators of the Wnt signaling pathway. For example, compound ICG-001 was shown to block the interaction between β-catenin and the transcriptional coactivator protein CREB binding protein (CBP) [50]. Recently, several new compounds that target tankyrase activity were also developed such as NVP-TNKS656 [51], G007-LK [52], and LGK974 [53]. Moreover, a small-molecule porcupine (PORCN) acyltransferase inhibitor, IWP-2, was found to inhibit the secretion of functional WNT ligands [13]. Here we identify repressors that inhibit *WNT1* expression through stabilizing the G-quadruplex structure formed at its promoter. The 6-substituted 9-chloro-11H-indeno[1,2-*c*]quinolin-11-one derivatives are capable of inhibiting the *WNT*-signaling pathway through suppressing the expression of *WNT1*. Thus, our results support a unique approach to inhibit *WNT*-mediated signaling pathway through suppression of *WNT1* expression. The identified target compounds should have potential to be further developed into drugs to treat Wnt-related diseases.

The cell-based reporter assay we established enables efficient monitoring and analyzing *WNT1* expression. In our screen system, we have also added *WNT1*-m1 and -m6 mutation constructs to enable the determination of whether the repression requires G-quadruplex structure formation. Thus, this cell-based system could be applied to gauge the expression of *WNT1* in a G-quadruplex structure dependent manner. Using the reporter system, we have successfully identified SJ26. SJ26 effectively represses the expression of *WNT1*, its downstream signaling pathway, and the migration activity of cancer cells. Importantly, the *WNT1* inhibitory effect mediated by SJ26 appears to be selective and specific as it does not affect the general proliferation property of cells at concentrations as high as 30 μM. It is also interesting to note that SJ26 only moderately elevates the melting temperature of a G-quadruplex structure formed by *WNT1* promoter sequences. Although moderate, the melting-temperature elevation is sufficient to repress *WNT1* expression and inhibit its downstream signaling pathway. Thus, the results suggest that our reporter assay could identify G-quadruplex stabilizers with high sensitivity.

Azafluorenone compounds show diverse pharmacological activities. For example, they have been reported to have antifungal, antimicrobial, and antimalarial activities [54–56]. They were also shown to have cytotoxic properties to induce cancer cells into apoptosis [57–59]. Moreover, an arylindenopyrimidine was shown to be a potent dual A2A/A1 receptor antagonist that might have the potential for the treatment of neurodegenerative disorder such as Parkinson's disease [60]. Our results in stabilization of the G-quadruplex structure point to a new pharmacological direction that azafluorenone compounds could also target to specific DNA structure. Much work yet needs to be done to fully understand the precise mechanisms of these series compounds. In conclusion, we demonstrated that SJ26 play a novel anti-cancer mechanism, and it has the potential to be further developed into anti-cancer drugs that targets the migration activity of cancer cells.

## MATERIALS AND METHODS

### Chemicals and test derivatives

In this study, we systematically synthesized tetracyclic and heterocyclic pharmacophores by introducing a series of side chains linked to the 9-chloro-11*H*-indeno[1,2-*c*]quinolin-11-one moiety. The synthesis of 3,6-bis(1-methyl-4-vinylpyridinium)carbazole diiodide (BMVC) has been described previously [61]. General chemicals used in this study were purchased from (Sigma-Aldrich). Cell lines (human lung cancer H1299; human breast cancer MCF7; human liver cancer Hep3B 2.17) were obtained from the Bioresource Collection and Research Center in Taiwan. All reactions were monitored by TLC (silica gel 60 F_254_). ^1^H NMR and ^13^C NMR were measured on Varian GEMINI-300 (300 MHz) or Agilent 400 MR DD2 (400 MHz); δ values are in ppm relative to TMS as an internal standard. Multiplicities are recorded as s (singlet), d (doublet), t (triplet), q (quartet), quin (quintuplet), dd (doublet of doublets), dt (triplet of doublets), td (doublet of triplets), m (multiplet), and br (broadened). Mass spectral analyses were conducted using high resolution electron impact ionization (HREI, Finnigan MAT MAT-95XL) in the Instrumentation Center of National Tsing-Hua University, Hsinchu, Taiwan. Melting points of synthetic compounds were determined with a Büchi B-545 melting point apparatus. The purity of all compounds was analyzed on a C18 reverse-phase column (XBridge BEH Shield RP18 Column, 130Å, 5 μm, 4.6 mm X 250 mm, Waters) by HPLC (model L-2000, HITACHI) with UV detection (model L-2400, HITACHI). Compound was dissolved in MeOH; the mobile phase was water and MeOH. A preliminary evaluation of the UV spectra was carried out by spectrophotometric analysis to determine the value of λmax for each compound. The purities of the synthetic compounds for biological evaluation were greater than 95%. Reagents and solvents were purchased from Merck and Sigma Aldrich and used without further purification. Typical experiments illustrating the general procedures for the preparation of the compounds are described below.

### Cell-based assay for monitoring *WNT1* expression

The *WNT1* promoter ranging from −600 to 201 relative to the transcription starting site was PCR-amplified from H1299 genomic DNA and cloned upstream to a secreted alkaline phosphatase (SEAP) reporter gene to generate pWNT1-SEAP [19]. This DNA fragment contains the G-quadruplex forming sequences and the *cis*-regulatory elements of *WNT1* transcription. To construct a mutant that fails to form G-quadruplex structure, plasmid pWNT1-SEAP was used as the template for mutagenesis using *Pfu* DNA polymerase (Stratagene). The resulting mutations were verified by DNA sequencing of the plasmids. These plasmids were introduced into cancer cell line H1299 and the stable cell lines harboring these plasmids were then isolated. Compared to their parental H1299 cell, no apparent change in the morphology or growth rate was observed in these stable lines.

### SEAP assay [62]

Secreted alkaline phosphatase was used as the reporter gene to monitor the transcriptional activity of *WNT1.* About 2 × 10^4^ each of cells were grown in 96-well plates and incubated at 37°C for 24 h and then changed with fresh media. Indicated amounts of drugs were added and then incubated for another 24 h. The culture media were collected and heated at 65°C for 10 min to inactivate heat-labile phosphatases. To assay phosphatase activity. an equal amount of SEAP buffer (2 M diethanolamine, 1 mM MgCl_2_, and 20 mM L-homoarginine) was added to the media, and *p*-nitrophenylphosphate was added to a final concentration of 12 mM. Absorptions at 405 nm were taken (BECKMAN BIOMEK 3000), and the rate of absorption increase was determined.

### MTT (3-(4,5-dimethylthiazol-2-yl)-2, 5-diphenyltetrazolium bromide) assay

Cells were first grown in 96-well plates (∼2000 cells per well) in the presence of 5% CO_2_ at 37°C. To examine the cytotoxic effect of the tested compounds, cells were incubated with different concentrations of compounds for 24 h. The cytotoxicity was then determined by adding MTT solution to cells to a final concentration of 0.45 mg/ml and incubated at 37°C for 1 hour. The resulting formazan crystals was dissolved and analysed spectrophotometrically at the absorbance of 570 nm.

### DNA preparation

All oligonucleotides were purchased from Bio Basic (Ontario, Canada) and used without further purification. DNA samples were prepared by dissolving oligonucleotides in 10 mM Tris-HCl (pH 7.5). DNA concentrations were determined using a UV-visible absorption nanophotometer (Implen). KCl at 5 mM was added to the DNA samples 70 min before experiments. For SJ26 treatments, the DNA samples were incubated with SJ26 for 30 min before experiments.

### Circular dichroism (CD) spectra and melting temperature

The CD spectra were recorded using a spectropolarimeter (J-815, Jasco, Japan) with a bandwidth of 2 nm at a scan speed of 50 nm/min and a step resolution of 0.2 nm under N_2_ over the spectral range of 210–350 nm to monitor the G4 structures. Thermal melting curves were recorded by a Peltier thermal coupler chamber (PFD-425S/15, Jasco) and the molar ellipticity was monitored at 265 nm between 10 and 100°C with a temperature ramping rate of 1°C/min rate. The melting temperature (*Tm*) was measured from the first differential of the melting curve.

### Quantification of *WNT1* RNA

The real-time quantitative RT-PCR was also used to determine the mRNA of *WNT1*. Cells were treated with SJ26 for 1 day and then subjected to RT-PCR analysis. Total RNA was isolated using TRIzol reagent (Sigma) and reverse-transcribed by random hexamers using cDNA reverse transcription kit (Applied Biosystem). The reverse-transcribed products were then analyzed using Cyber Green I system (FastStart Universal SYBR Green Master, Roche Applied Science). Primers used for PCR reactions were: WNT1 (forward primer 5′-CTGTCCTGCCTCCTCATC-3′ and reverse primer 5′-GGACCCAGCACAATAAATAGTT-3′); GAPDH (forward primer 5′-TAACTCTGGTAAAGTGGATA-3′ and reverse primer 5′-AAGATGGTGATGGGATTT-3′). The parameter Ct is defined as the fractional cycle number at which the fluorescence is generated by the Cyber green I system. Quantifications were first determined by normalizing the Ct values of the tested mRNA with the Ct of *GAPDH*. The relative values were then obtained using no drug treatment as 100%. Quantification results were obtained from three independent experiments.

### Immunoblotting analysis

The SJ26-treated H1299 cells were washed twice with phosphate-buffered saline (PBS) and lysed using radioimmune precipitation assay buffer (10 mM Tris-HCl (pH 7.4), 150 mM NaCl, 5 mM EDTA, 1% Triton X-100, 100 μM phenylmethylsulfonyl fluoride, 1 mg/ml leupeptin, 1 mg/ml aprotinin, and 25 mM dithiothreitol). Equal amount of total proteins were separated on SDS-PAGE gels and transferred to nitrocellulose membranes. Antibodies against Wnt1 (1:1000 dilution, Spring, REF E3960), β-catenin (1:1000 dilution, GeneTex, GTX61089), and α-tubulin (1:5000 dilution, Millipore, MAB374) were used as the primary antibodies. Horseradish peroxidase-conjugated anti-mouse IgG or anti-rabbit IgG (Amersham Biosciences) were used as the secondary antibodies. Blots were visualized using enhanced chemiluminescence system (PerkinElmer Life Sciences).

### Scratch assay

Cells were treated with compound SJ26 for 1 day and subjected to scratch assay. The drug treated cells were plated and scratched with a pipette tip to generate a cell-free zone. Cells were then incubated at 1% serum culture medium to avoid further cell proliferation. Migration of cells toward the cell-free zone was monitored after 24 h using a Leica DFC 420c camera (Leica Microsystems), and the migration rates were determined. The relative healing activities were obtained using the value of solvent control as 100%.

### Trans-well assay

About 2 x10^5^ of the drug-treated cells were grown in the inner chamber of Millicell Hanging Cell Culture Insert (pore size 8.0 μm, Merck Millipore) in a serum-free medium and with 10% serum medium in the outer chamber. The cells were incubated at 37°C for 6 h to allow the migration toward the outer side of the inner chamber. Cells were fixed with 4% paraformaldehyde for 2 min, and then permeabilized with methanol for 20 min. The cells on the inner layer were softly removed with a cotton swab, and the adherent cells on outer surface were stained with 0.3% crystal violet dye for 15 min and then counted. The relative migration activities were obtained using the value of solvent control as 100%.

### Statistical analysis

Student's *t*-test was applied to assess whether the means of two groups are statistically different from each other. Here, we consider *P* < 0.05 as significant.

## Abbreviations

WNT1: wingless-type MMTV integration site family, member 1
CD: circular dichroism
Tm: melting temperature
SEAP: secreted alkaline phosphatase
SARs: structure-activity relationships
CPT: Camptothecin
Topo: topoisomerase
BMVC: 3,6-bis(1-methyl-4-vinylpyridinium)carbazole diiodide
CD: circular dichroism
H1299: human non-small cell lung carcinoma cell
HRMS: high resolution mass
PBS: phosphate-buffered saline
DMSO: dimethyl sulfoxide
THF: tetrahydrofuran
TEA: triethylamine.
